# Correction: MUC1-C represses the RASSF1A tumor suppressor in human carcinoma cells

**DOI:** 10.1038/s41388-019-1038-5

**Published:** 2019-10-01

**Authors:** Hasan Rajabi, Tsuyoshi Hata, Wei Li, Mark D. Long, Qiang Hu, Song Liu, Deepak Raina, Ling Kui, Yota Yasumizu, Deli Hong, Mehmet Samur, Donald Kufe

**Affiliations:** 1 Dana-Farber Cancer Institute, Harvard Medical School, Boston, MA USA; 2 Department of Biostatistics & Bioinformatics, Roswell Park Comprehensive Cancer Center, Buffalo, NY USA; 3 Present Address: Genus Oncology, Boston, MA USA

**Keywords:** Oncogenes, Cancer genomics

**Correction to: Oncogene**

10.1038/s41388-019-0940-1

The original version of this Article contained an error in the spelling of the author Yota Yasumizu, which was incorrectly given as Yota Yazumizu. This has now been corrected in both the PDF and HTML versions of the article.

